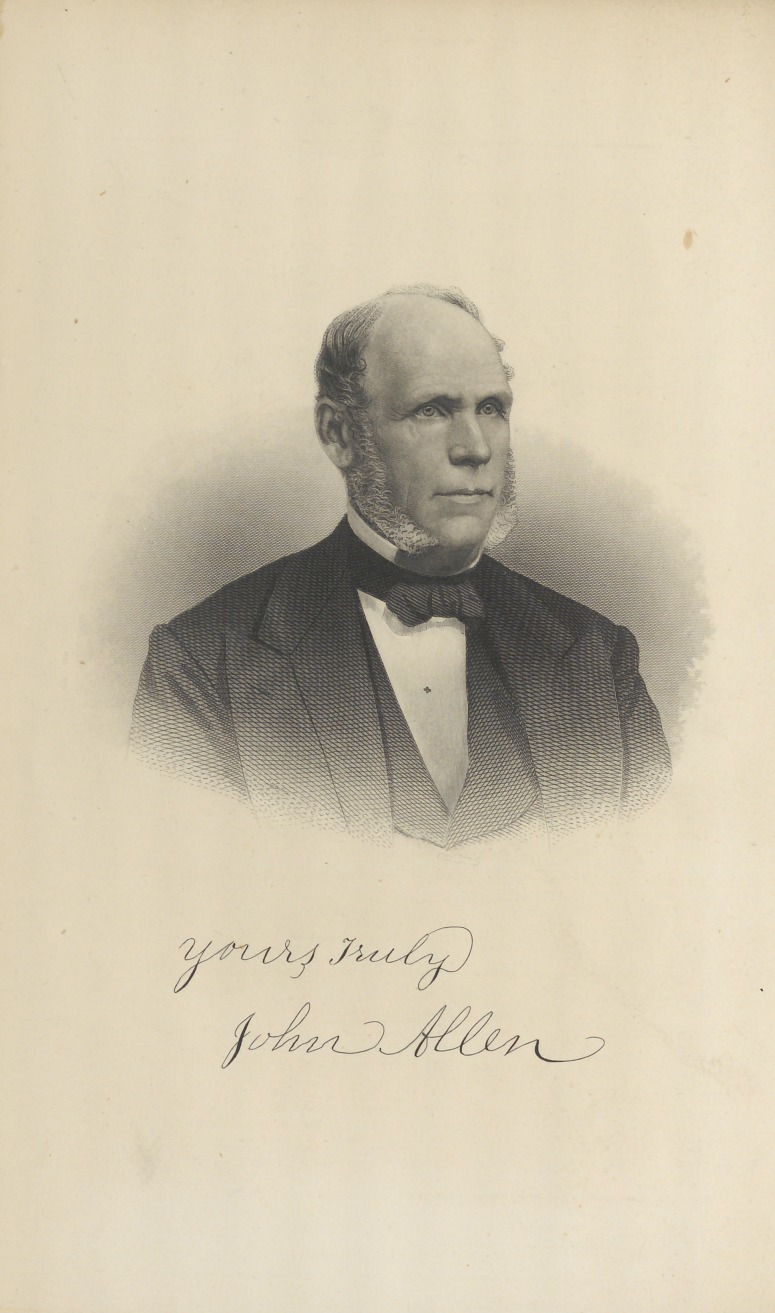# A Biographical Sketch of Dr. John Allen

**Published:** 1874-04

**Authors:** 


### DENTAL REGISTER.

VOL. XXVIII.] APRIL, 1874. [NO. 4.

### A BIOGRAPHICAL SKETCH OF DR. JOHN ALLEN, OF NEW YORK.

Dr. John Allen was born in Broom county, New York, Nov. 4, 1810. He was a descendent of the Ethan Allen family, of Vermont, whose history is identified with the Revolutionary War. His father (Dr. N. Allen) moved to Ohio when John was but a small boy. Here, the allurements of agriculture engrossed the attention of the family for several years. At the age of nineteen this son became a student of Dr. James Harris, a medical man of high standing, who relinquished the practice of medicine for that of dental surgery. At that time, practitioners in this specialty in the West were comparatively few, and a large field for usefulness was opened before him, in which he zealously devoted his time and talent, the remainder of his life which was only a few years; but his "works live after him."

After having concluded his course of pupilage with Dr. Harris, he commenced his professional career in Cincinnati, in 1830. Here in the early part of his practice, with time on his hands, he availed himself of the Medical College of the city for acquiring a more thorough foundation for the practice of this specialty. Being the only dentist in the college, it was agreed by the other students that when subjects were obtained for dissection he should be entitled to the teeth for subsequent use in his practice. This was quite an item for him at that time, as good human teeth were then worth one dollar a piece. In preparing them for use, the crowns of the teeth were cut off from the roots and the pulps removed; they were then preserved in spirits of wine. The teeth of cows and other animals were also obtained and preserved in like manner to be used as substitutes for the natural teeth.

Carved dentures from the hippopotamus were also used for this purpose, as porcelain had not then been brought into practical use., although many fruitless experiments had been made, both in France and in this country.

When the practicability of mineral teeth as artificial substitutes became established; Dr. Allen sought and obtained a practical knowledge of their manufacture, that he might be better able to meet the requirements of the various cases which occur in dental practice. Single and block teeth, when well mounted upon gold plates, were considered the highest style of artificial dentistry that had then been attained. But still there were defects that even this method had failed to overcome. And although he had reached the maximum in the production of this style of work, yet the seams and fissures, the stiff, mechanical appearance, and in many instances a failure to restore the natural form and expression of the mouth and face, were serious obstacles yet to be removed, and to rest satisfied with such an imperfect method was to stop short of what was required for artificial dentistry.

To meet this apparent demand for some mode by which more perfect results could be obtained, Dr. Allen resolved to commence various experiments with a view of working out a new system which he had conceived, which was yet vague and chaotic, a mere germ. But the how to develop his system led through a dark and tangled way, along an untrodden path, with no light but that which he had made for himself as he advanced towards the goal where he had placed his mark.

His first steps in this direction were to test the practicability of raising the sunken portion of the face, in cases where the original form and expression had become sunken or changed by the loss of teeth and consequent absorption of the alveolar processes.

This was a new feature in dental practice, for, as yet, there were no records to show that it could be done by artificial means without doing injury to the parts raised. This was a question he resolved to settle by thoroughly testing its possibility. The result of his efforts proved successful. He then brought the subject before the American Society of Dental Surgeons and clearly demonstrated to that body its practicability. The Society signified their appreciation of this contribution to dental science by awarding him a gold medal with the following inscription: upon one side is a beautiful engraving, representing the temple of science with a light upon the top reflecting its rays in all directions. To this device the following sentence is prefixed: "Societas Americana Qui Dentium Vitia Curant." Upon the other side are these words, "Awarded to Dr. John Allen for his invention for restoring the contour of the face, Aug. 1843."

This question being settled for all future time, his next efforts were directed to the working out of his conceived idea of a process by means of which to overcome the defects that existed in what was called plate work. As teeth were then mounted on metalic plates, three diffent parts or substances were employed, viz: the teeth, the plate and the solder, but to accomplish his purpose, an other substance must be added in the form of a fusible flesh colored cement or enamel with which to form an artificial gum roof and rugæ of the mouth without seam or crevice. If this fourth substance could be obtained and properly adapted to dentures, a more truthful representation of the natural organs would be the result, as the teeth could be arranged either irregularly or symmetrically as different persons might require, and there would be no fissures or interstices for foul secretions to vitiate the saliva or infect the breath. These advantages he deemed of sufficient importance to justify the most earnest researches to attain. But to get this fourth substance out of chaos required various preparations of minerals, metals, oxides, pigments, fluxes, precipitates, etc., etc. These together with the exact proportion of each constituent necessary to produce the desired results were brought into requisition. This required much time, perseverance and expenditure, attended with successive series of experiments, ever varying with the quality, quantity and manipulations of the materials used.

Here was a large field for explorations, in which he could make haste slowly, as he had no borrowed light from any successful predecessor. Many of his earlier experiments were made upon gold plates, as they were considered the best for dental purposes that were then in use. But experience proved to him that an enamel that would flow upon a gold plate would not stand the secretions of the mouth, for the reason that too much flux was required in the compound in order to make it flow at a less heat than the melting point of gold, which is below two thousand degrees; consequently this line of experiments proved a failure.

Another series of experiments was then commenced, with plate; which was difficult to obtain, as no plate of this kind was then in the market for such a purpose. But it must be had, and was procured at a price corresponding with the scarcity and value of this metal. With this plate for a base, which no furnace heat would melt, and with new formulas of different preparations and proportions, a much harder, flesh-colored enamel was at length obtained, which could not be affected by the secretions of the mouth, and in point of appearance (with proper manipulation) produced results true to nature. This achievement, together with the one previously made by him for restoring the contour of the face, completed his system for constructing artificial dentures. This method combines four important advantages not previously obtained:

First.—By means of a beautiful flesh-colored enamel, the teeth are garnished with an artificial gum, roof and rugæ of the mouth (without seam or crevice), with all the delicate tints and shades peculiar to those of nature.

Second.—A truthful expression is given to the teeth by arranging them either symmetrically or irregularly, as the patient may require.

Third.—The sunken portions of the face can be restored by means of attachments or prominences, made upon the denture, of such form and size as to meet the requirements of each particular case.

Fourth.—No metal plate or unnatural appearing substance can be seen in the mouth of the wearer, when laughing, singing or yawning.

The Baltimore Dental College was the first one established and is still in a prosperous condition. It was organized in 1840. The faculty consisted of Drs. C. A. Harris, Tho. Bond and H. H. Haydon. The next was the Ohio Dental College, which was established in Cincinnati in 1845. Drs. James Taylor, Jesse W. Cook and M. Rogers constituted the faculty. This was preceded by the formation of the Mississippi Valley Dental Association, which was formed in 1844. The first charter of the College being defective, a new charter was formed by Dr. Allen, who went to Columbus, the seat of government of Ohio, and had it introduced in the Legislature; and he remained there to guard its interests until it passed both branches of that body and became a law. The College was then re-organized upon a firm basis, and, with a slight amendment of the charter. has continued as a permanent institution to the present time, owning, as it does, the College building, together with the requisite appurtenances necessary for the school.

In this institution Dr. Allen filled a chair for some years, with reference to which the Dental Register of the West makes the following allusion:

"Dr. J. Allen's Resignation and Removal—We learn that Dr. J. Allen, who has enjoyed the confidence and patronage of this community, as a dental practitioner for more than twenty years, is about establishing his business in the city of New York, with a view of directing his exclusive attention to his improved style of work.

"This change in his business operations has rendered it necessary for him to resign his professorship in the Ohio College of Dental Surgery, a chair which he has filled with *ability and general satisfaction; and we regret the necessity which impels him to leave the school.* Our wish is, that he may meet with many friends and great success in New York, where most of his time will be likely spent. We can commend him to our brethren in New York, as a gentleman devoted to the profession, courteous, affable and obliging."

This advance in dental prosthesis was the dawn of a new era in his profession, which induced him to remove to the city of New York for his future field of labor, although he had a large practice in Cincinnati at that time. This was in 1853. Here his son, Charles D. Allen, having returned home from his collegiate course, joined his father, first as a student and assistant, and finally as a partner in dental practice. This firm still continues in New York under the name of J. Allen & Son.

In order to fix the date of his improvement, and perhaps receive some remuneration for his years of toil and expenditure, Dr. Allen had it placed upon the national records in the American Patent Office and also in that of England, for which he received letters patent in both countries. But such was the feeling against dental patents (without discrimination) that he did not press his claims upon the profession, although in one or two instances he became involved in litigation in defense of his system.

The details of this method were such that very few dentists could execute the work without instructions, which were readily imparted by Dr. Allen to those who desired to introduce it in their practice, charging merely for the time and attention bestowed. There were others, who prferred sending their cases of this kind to him to be made, for which he charged only for the labor and materials used. In his professional career, he has ever maintained a high and honorable position, preferring to secure commendation by industry, faithfulness, honesty and kindness, as evinced, not only in his social relations, but also in the many essays, lectures and scientific contributions from him, which we find published through various dental, medical and other journals, embracing a period of more than thirty years. These, together with his code of dental ethics, (the first in the dental profession,) which he prepared and brought before the American Dental Association in Chicago, at the fifth annual meeting of that body, furnish the outlines of his professional course.

In tracing out the land-marks of Dr. Allen's professional career, we have merely leaped from point to point, touching only upon such points of his history as pertain to American dentistry; omitting the details of more than forty consecutive years of his dental practice, during which time he has received, not only many tokens of personal regard, for his services in operative dentistry, but also numerous official testimonials, in the form of professional diplomas, medals, reports, etc., some of which we will briefly notice. After perfecting his system of constructing artificial dentures, he was desirous of bringing it in fair competiton with all other modes, wrought by skillful dentists, both in this country and in Europe. As before stated, in 1845 he received a medal from the American Society of Dental Surgeons. In 1857, another was awarded to J. Allen & Son by the American Institute. In 1857, a medal was awarded to his son, C. D. Allen, for this style of work. In 1863, another medal was awarded to J. Allen & Son by the American Institute. In 1867, a magnificent medal was awarded to J. Allen & Son by the American Institute. In 1867, a much larger and more splendid medal was awarded by the World's Exposition, at Paris, to J. Allen & Son. In 1873, at the great World's Exposition at Vienna, another gold medal, still more elaborate than any of the preceding ones, (as it carried with it the Medal of Progress,) was awarded to J. Allen & Sons, of New York.

Yours Truly

John Allen

## Figures and Tables

**Figure f1:**